# The effect of QPQ treatment on the wear and corrosion resistance of 42CrMo bearing steel

**DOI:** 10.1038/s41598-025-24065-w

**Published:** 2025-11-17

**Authors:** Chengyuan Ni, Jingtao Yang, Chengdong Xia, Zhongyu Piao, Minghua Yin

**Affiliations:** 1 Zhejiang Provincial Key Laboratory of Intelligent Manufacturing for Aerodynamic Equipment, Quzhou, 324000 China; 2 College of Mechanical Engineering, Zhejiang University of Technology, Hangzhou, 310023 China; 3 Zhejiang Zhongcheng Sliding Bearing Technology Co., Ltd., Quzhou, 324000 China

**Keywords:** QPQ treatment, 42CrMo, Nitriding temperature, Nitriding time, Microstructural morphology, Surface properties., Engineering, Materials science

## Abstract

This study investigates the impact of QPQ (Quench–Polish–Quench) treatment parameters—580 °C and 620 °C, with holding times of 90 min and 120 min—on the microstructure, wear resistance, and corrosion resistance of 42CrMo bearing steel. Microstructural evolution was analyzed by OM, SEM, and XRD, while hardness, friction-wear, and electrochemical tests assessed performance. QPQ treatment produced a three-layer surface structure comprising an oxide film, a compound layer rich in Fe₂₋₃N and Cr₂N, and a diffusion layer. At 620 °C for 120 min, the compound layer reached a thickness of 24.01 μm, with a diffusion depth of 377 μm, and the surface hardness increased to 710.9 HV_0.2_. The lowest friction coefficient (0.33) and wear loss were observed for the 620 °C × 90 min sample, whereas the highest corrosion resistance occurred for the 580 °C × 120 min sample (E_corr_ = − 0.476 V, log_10_(I_corr_) = − 6.242, R_p_ = 2643 Ω). Temperature exerted a more substantial influence on layer growth and nitrogen diffusion than holding time. However, prolonged treatment at 620 °C resulted in spallation, embrittlement, and porosity, thereby degrading the wear and corrosion performance.

## Introduction

Surface wear and wear-induced failure of mechanical components are significant causes of equipment accidents^1^. Such failures often occur in parts operating under moderate speeds and loads, such as automotive steering knuckles, rear axles, gears, and shafts. Under the “Made in China 2025” initiative, higher performance demands have been placed on bearings operating in extreme service environments—characterized by elevated temperatures and heavy loads—thereby underscoring the need to improve wear resistance, corrosion resistance, load-bearing capacity, fatigue life, and environmental adaptability^2–4^. An ideal bearing material should integrate excellent friction-reducing ability, wear resistance, and anti-seizure properties with excellent frictional conformity, run-in behavior, sufficient mechanical strength, and corrosion resistance^5–7^. Various bearing materials have been developed worldwide, including Babbitt alloys, copper alloys, aluminum alloys, iron-based alloys, and TiAl alloys. Babbitt alloys offer excellent anti-friction properties but have low load capacity and poor heat and fatigue resistance^8–10^. Copper alloys provide superior strength and thermal performance but are costly^11,12^. By contrast, iron-based alloys combine high compressive strength, self-lubrication, and low cost, and are the most widely used bearing materials^13^.

42CrMo bearing steel, a widely used medium-to-high-strength alloy steel, is typically subjected to quenching and tempering to achieve high strength, superior toughness, and outstanding hardenability. However, due to frequent exposure to friction and corrosive media during service, its wear and corrosion resistance are insufficient, restricting its use to light-load, low-speed friction components. Moreover, the relatively low chromium content at the surface limits the formation of a stable passivation film, further diminishing its corrosion resistance. Therefore, enhancing the surface wear and corrosion resistance of 42CrMo bearing steel is crucial for improving overall stability and extending service life. Surface coating technology has advanced rapidly in recent years. Numerous scientific studies demonstrate that coatings can significantly improve the friction properties of mechanically interacting surfaces.

Wang and Duan^14^ used laser cladding to create Cr–Si–Ni composite coatings on austenitic stainless steel (1Cr18Ni9Ti) and examined the connection between the microstructure and properties of the coatings and the volume fraction of Cr₃Si. They discovered that increasing the Cr₃Si content improved hardness, corrosion resistance, and wear resistance. Bobzin et al.^15^ deposited carbon- and chromium-based coatings on cylindrical roller thrust bearings using physical vapor deposition (PVD) and demonstrated that the coatings reduced wear and friction. Low-temperature nitriding introduces nitrogen at reduced temperatures to form a thin, hard compound/diffusion layer without compromising the quenched matrix. This process has been widely reported to improve surface hardness, wear resistance, and fatigue crack initiation thresholds in bearing steels^16^.

QPQ technology offers several advantages under high-speed and heavy-load conditions compared with low-temperature nitriding, hard coatings (e.g., DLC/PVD), and laser cladding. These advantages include superior wear and hardness, enhanced fatigue strength, improved corrosion resistance, excellent dimensional stability, applicability to complex geometries, and greater cost- and time-efficiency. In addition, the process is environmentally friendly due to the absence of cyanide, making it suitable for high-performance components in construction machinery, automotive applications, and tooling^17–19^. QPQ temperature and duration are key parameters governing the nitrogen diffusion rate and the quality of the compound layer. According to the Fe–N binary phase diagram, nitriding above 590 °C promotes the formation of nitrogen-containing austenite; thus, 590 °C is considered the critical temperature separating ferritic from austenitic nitriding^18^.

Bonow et al.^20^ added KCl to the nitriding salt, preparing a mixture of 75% potassium nitrate and 25% potassium chloride. QPQ treatment at 650 °C for three hours was applied to low-carbon steel (0.2% C), resulting in a 3.5-fold increase in hardness, reaching 702 HV. Guo Jie et al.^21^ nitrided 65Mn steel at 570 °C for three hours, increasing surface hardness to 700 HV_0.1_ and reducing wear loss to 18.8% of that of the base material. Fu et al.^22^ raised the nitriding temperature of hot-work die steel from 540 °C to 560 °C, producing a more uniform compound layer and increasing Fe₃O₄ formation on the surface. Compared with untreated samples, the corrosion potential increased by 200 mV, and the corrosion current density decreased by two orders of magnitude, thereby enhancing corrosion resistance; however, the impact toughness was significantly reduced. Tang Cai et al.^23^ performed QPQ treatment on 40Cr steel at 580, 600, 620, and 640 °C for five hours and identified 620 °C as the optimal temperature, yielding a nitriding layer depth of 0.29 mm and the lowest wear loss. Alberto Campagnolo et al.^24^ treated 39NiCrMo3 steel at 580 °C, observing increased brittleness but improved fatigue strength. Song Xiaoming^25^ subjected G80Cr4Mo4V to QPQ treatment at 520 °C for one hour, four hours, and eight hours, during which a porous surface layer formed, markedly increasing surface roughness. With longer nitriding times, slight increases in surface straightness and roughness were observed, which affected dimensional accuracy and surface finish. Wang Qinjuan^26^ found that when alloy cast iron was QPQ-treated at 580 °C, surface hardness decreased from 522 HV_0.05_ to 441 HV_0.05_ as nitriding time increased from 90 min to 150 min. Xiang^27^ reported that the nitrided layer thickness increased markedly when QPQ temperature exceeded 590 °C (reaching 41 μm at 610 °C). However, coarsening of the precipitated phases in the diffusion layer occurred, with the CrN morphology changing from spherical to rod-like, and the thickening of the porous layer led to a reduction in hardness.

Under high-speed or heavy-load conditions, 42CrMo bearing steel requires excellent wear resistance, fatigue resistance, and high surface strength. Although QPQ technology has been extensively studied, specific knowledge gaps regarding 42CrMo bearing steel compared to other alloys remain insufficiently elucidated. The correlations between process parameters, microstructural evolution, and service performance have not yet been fully clarified. The surface properties of 42CrMo are susceptible to variations in QPQ process parameters, which may limit its performance under severe service conditions. In this study, 42CrMo bearing steel was used to investigate the effects of different QPQ treatments on microstructure, hardness, wear resistance, and corrosion resistance. The mechanisms by which QPQ processing influences wear and corrosion behavior were analyzed, and process parameters were optimized to obtain bearing materials with superior overall properties. The findings aim to support the development of high-performance bearings for construction machinery applications.

## Result

### Microstructure

**Fig. 1:**
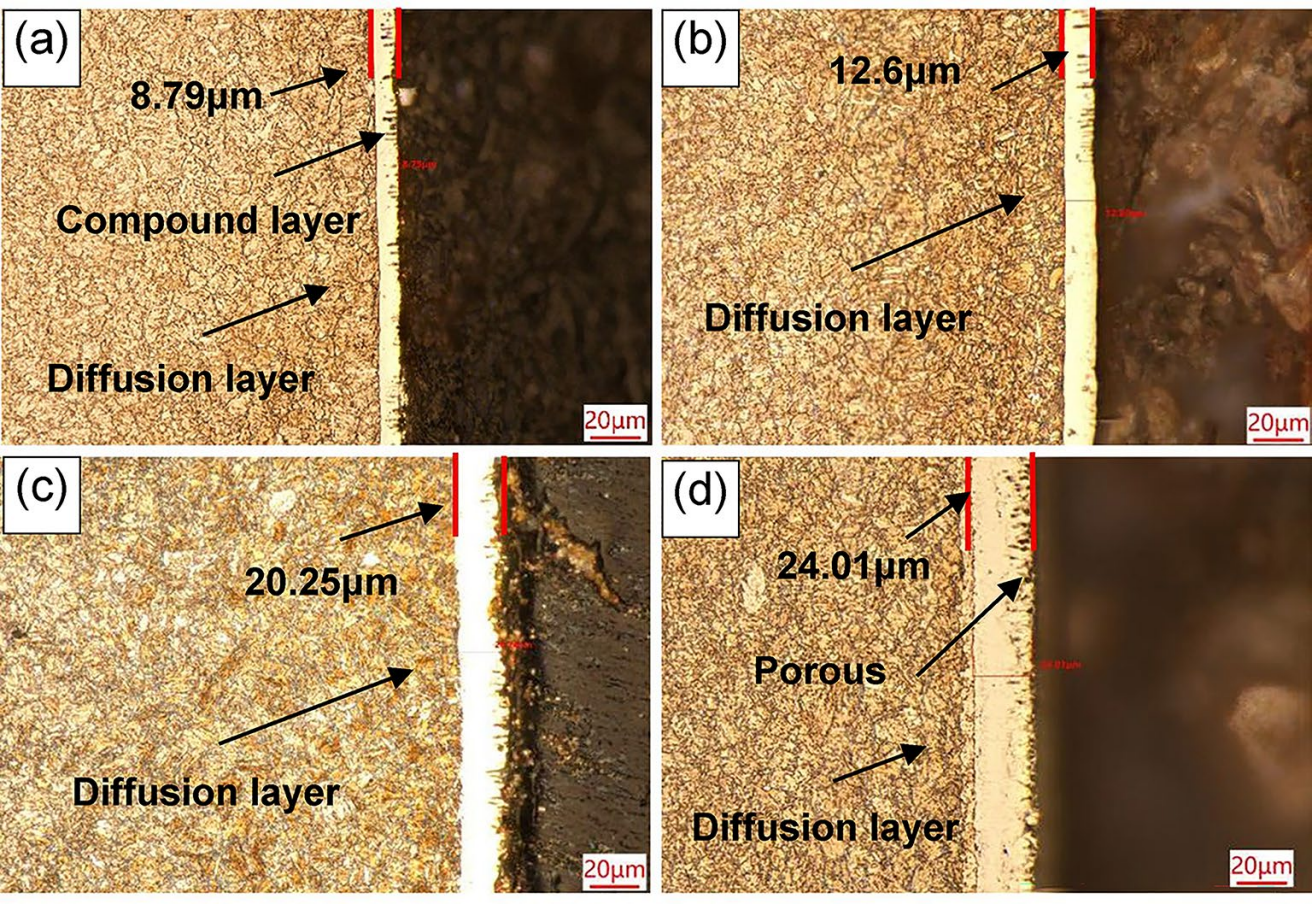
Cross-sectional microstructures of 42CrMo under different processes: (**a**) 580℃×90 min; (**b**) 580℃×120 min; (**c**) 620℃×90 min; (**d**) 620℃×120 min. Magnifications: 500×. The outermost thin oxide layer is difficult to observe under an optical microscope.$$\:\mu\:m$$$$\:\mu\:m$$ $$\:\mu\:m$$

**Fig. 2:**
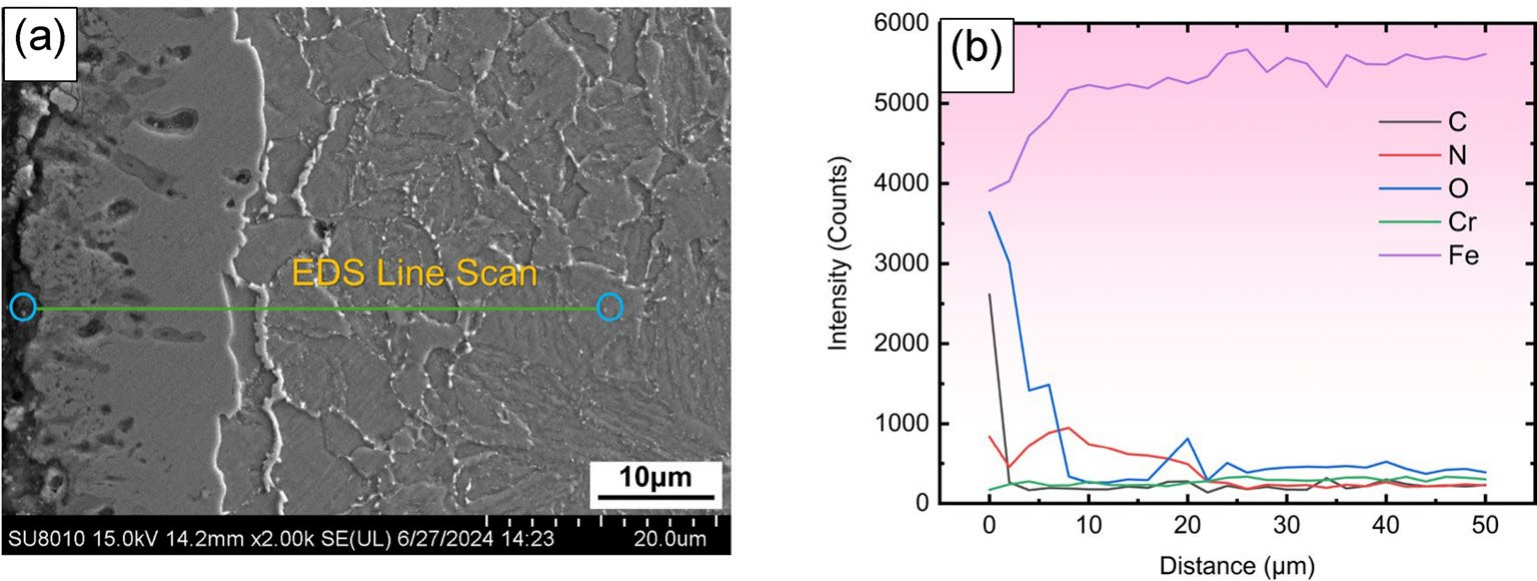
(**a**) EDS line scan region; (**b**) line scan results. As shown in (**b**), the surface has a high oxygen content, which confirms the presence of an oxide layer. Nitrogen content decreases as the distance from the surface increases.

The microstructural morphology of the QPQ specimen cross-section is depicted in Fig. 1. The outermost layer is a relatively thin oxide layer, which is difficult to observe under an optical microscope. However, the line scan results in Fig. 2 reveal a high oxygen content on the surface, confirming the presence of this oxide layer. The white regions correspond to the compound layer, while the adjacent dark region differs from the matrix and forms the diffusion layer. The compound layer thicknesses, measured and listed in Table 1, reached 8.79 $\:\mu\:m$ and 12.6 $\:\mu\:m$ after 90 and 120 min of nitriding at 580 °C, respectively. At 620 °C, the compound layer thickness increased to 20.25 $\:\mu\:m$ and 24.01 $\:\mu\:m$ after 90 and 120 min, respectively. Temperature has a more pronounced effect on thickness growth than time; increasing the temperature from 580 °C to 620 °C increased the thickness by 11.4 $\:\mu\:m$, whereas extending the time from 90 to 120 min increased only 3.8 $\:\mu\:m$.

**Table 1:** Compound layer thickness, nitrided depth, and diffusion coefficient for different QPQ processes.

Process	Compound layer thickness /	Pore area/	adequate nitriding layer depth /	Diffusion coefficient（m^2^/s）(×10^-12^)
580℃×90 min	8.79	334.96	284.8	4.53
580℃×120 min	12.6	218.95	289.3	
620℃×90 min	20.25	282.95	293.8	9.25
620℃×120 min	24.01	683.22	374.6	

**Fig. 3:**
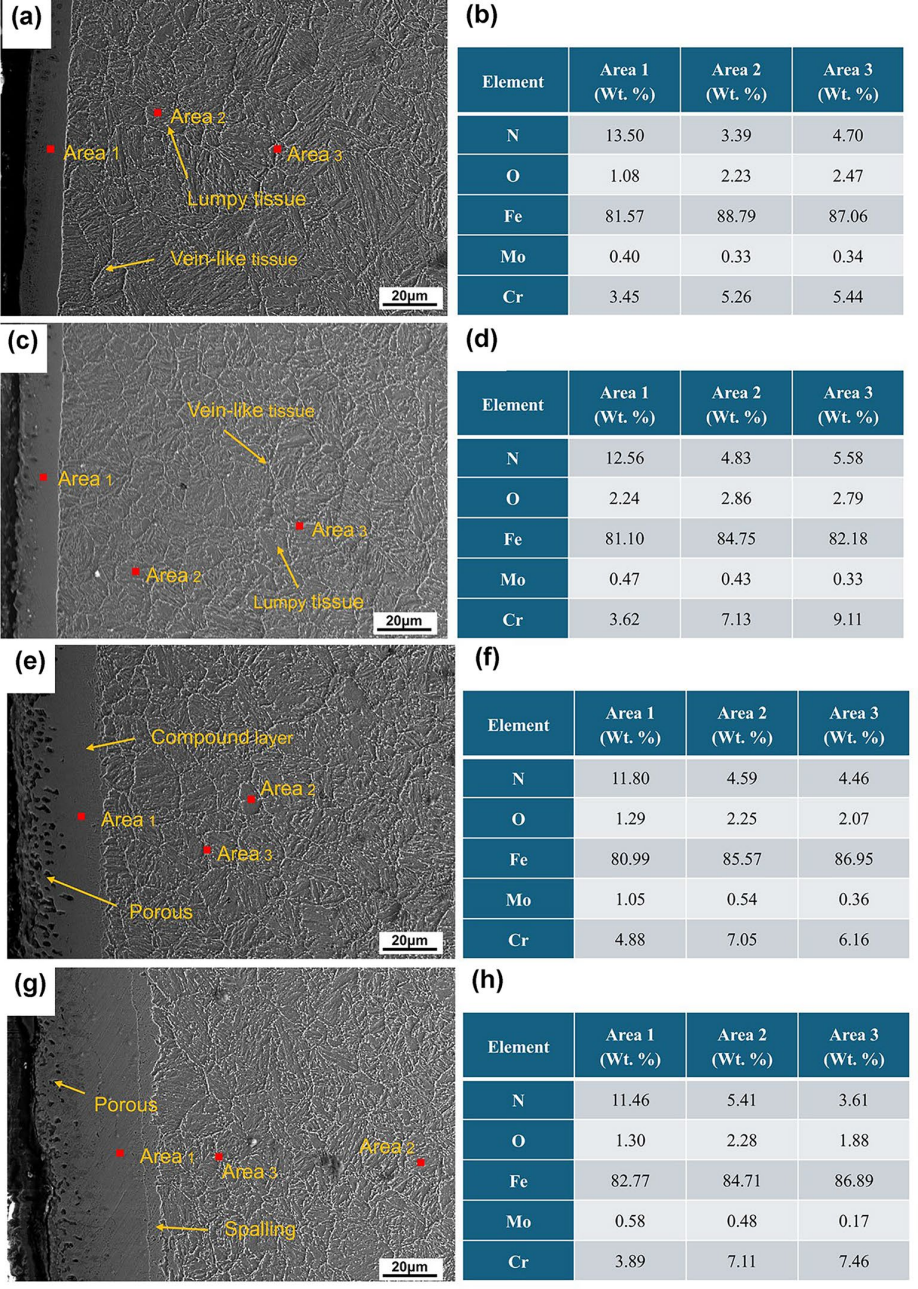
SEM images and EDS analysis of samples under different processing conditions: (**a**) 580 °C × 90 min, (**b**) EDS results at 580 °C × 90 min, (**c**) 580 °C × 120 min, (**d**) EDS results at 580 °C × 120 min, (**e**) 620 °C × 90 min, (**f**) EDS results at 620 °C × 90 min, (**g**) 620 °C × 120 min, and (**h**) EDS results at 580 °C × 90 min. Magnification: 500×.

Scanning electron microscope (SEM) images of the QPQ-treated samples are illustrated in Fig. 3. A pronounced porous structure was observed within the compound layer at 620 °C. Localized spalling of the compound layer was also detected under the 620 °C for 120 min (620 °C × 120 min) treatment condition. Two primary morphologies of compounds were identified in the diffusion layer: one exhibiting a plate-like structure and the other precipitating along grain boundaries in a vein-like distribution. According to the literature^28^, CrN, Cr₂N, γ′-Fe₄N, and ε-Fe₂₋₃N commonly coexist in nitrided layers, and their morphologies—such as layered, lamellar, and granular—depend on temperature and time.

The nitrogen content in the compound layer is considerably higher than that in the diffusion layer, as indicated by the EDS analysis results (Fig. 3). The compound layer also contains minor amounts of chromium (Cr) and molybdenum (Mo), and is primarily composed of Fe–Cr nitrides. In the diffusion layer, the Cr content within the vein-like precipitates exceeds the nitrogen content, where Fe₄N and Cr₂N precipitates are present. Block-like structures appear adjacent to these vein-like precipitates, exhibiting similar N and Cr contents. These structures are formed through the outward diffusion and aggregation of Cr₂N. As the temperature increases, the Cr and Mo contents in the compound layer rise, indicating the accumulation of Cr and Mo nitrides. However, prolonging the treatment time results in a smaller increase in Cr and Mo content compared to the effect of temperature. At 620 °C for 120 min, extended treatment causes nitride decomposition and escape, resulting in a decrease in Cr and Mo contents.

## Microhardness

**Fig. 4:**
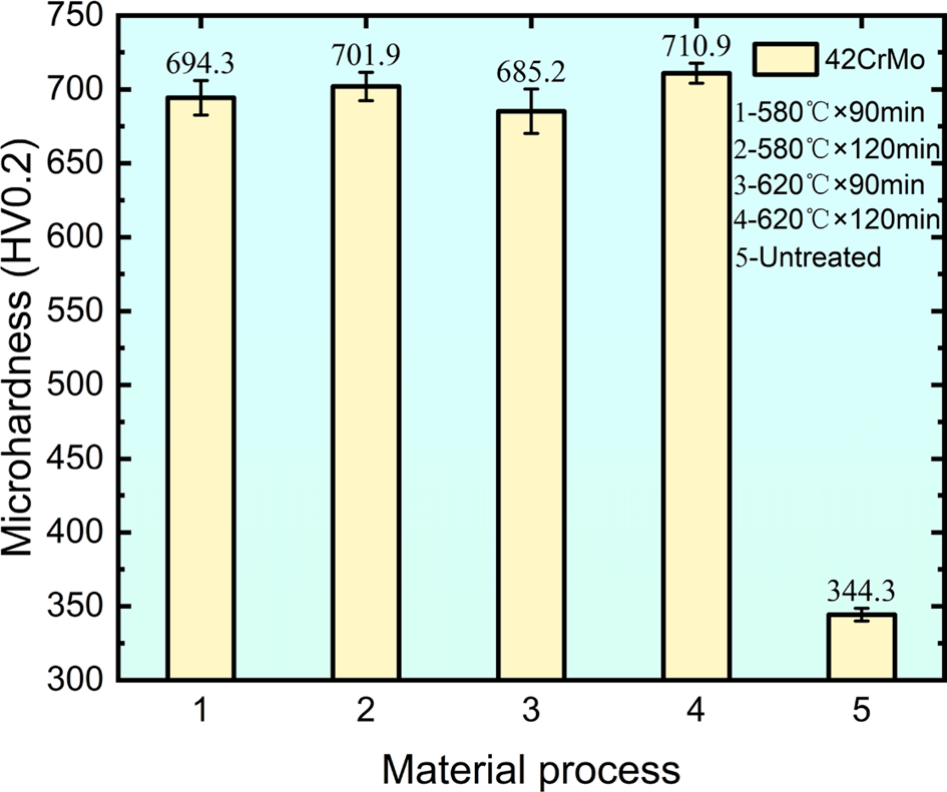
Surface hardness of different QPQ-treated samples: 1-580°C × 90 min, 2-580°C × 120 min, 3-620°C × 90 min, 4-620°C × 120 min, 5-Untreated.

The surface hardness of 42CrMo steel was substantially enhanced by QPQ treatment, as illustrated in Fig. 4. After holding at 580 °C for 90 and 120 min, the hardness reached 694.3 HV_0.2_ and 701.9 HV_0.2_, respectively. At 620 °C, the hardness was 685.2 HV_0.2_ and 710.9 HV_0.2_ for the same holding times. Compared with the untreated sample (344.3 HV_0.2_), the surface hardness increased by more than twofold. Notably, as the temperature increased from 580 °C to 620 °C, the hardness decreased slightly; for example, at 90 min, hardness dropped from 694.3 HV_0.2_ to 685.2 HV_0.2_. At a constant temperature, increasing the holding time led to increased hardness, as demonstrated by the rise from 685.2 HV_0.2_ to 710.9 HV_0.2_ at 620 °C when the time was extended from 90 to 120 min.

**Fig. 5:**
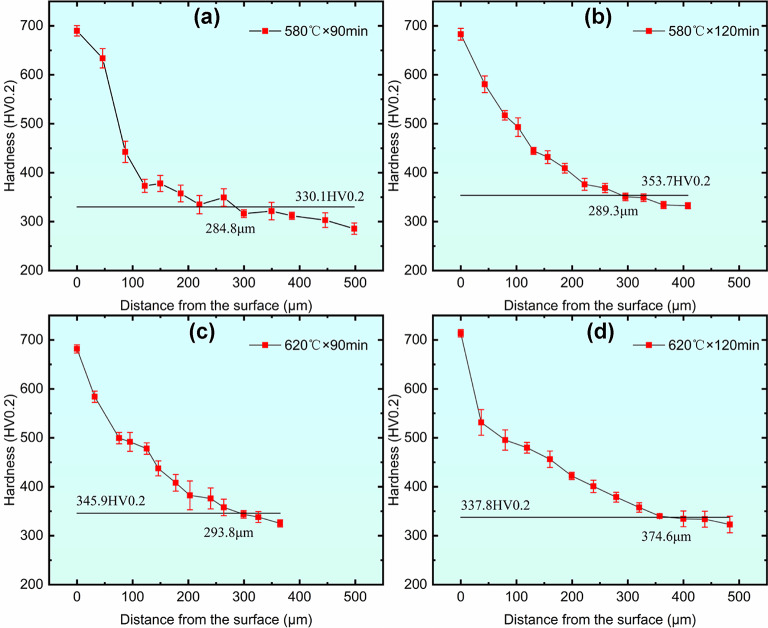
The hardness gradient is distributed across the cross-section of specimens undergoing different QPQ treatment processes. (**a**) 580℃×90 min, (**b**) 580℃×120 min, (**c**) 620℃×90 min, (**d**) 620℃×120 min.

As shown in Fig. 5, the cross-sectional hardness decreased overall from the surface toward the core but remained higher than the substrate hardness at a depth of 300 $\:\mu\:m$ from the surface. According to GB/T 11,354 − 2005, the adequate nitriding layer depth is defined as the position where hardness exceeds the substrate hardness by 30 HV. Based on this criterion, the nitrided layer depths under different QPQ treatments were calculated and are listed in Table 1. The effective nitriding layer depths were 284.8 $\:\mu\:m$ and 289.3 $\:\mu\:m$ for the 580 °C × 90 min and 580 °C × 120 min treatments, respectively. Similarly, the depths were 293.8 μm and 374.6 $\:\mu\:m$ for the 620 °C × 90 min and 620 °C × 120 min treatments, respectively. At a holding time of 90 min, the temperature had a negligible effect on the nitrided layer depth. At 620 °C, the layer depth increased gradually with extended holding time. As the temperature increased, the influence of prolonged holding time on the adequate nitrided layer depth became more pronounced.

## Friction and wear

**Fig. 6:**
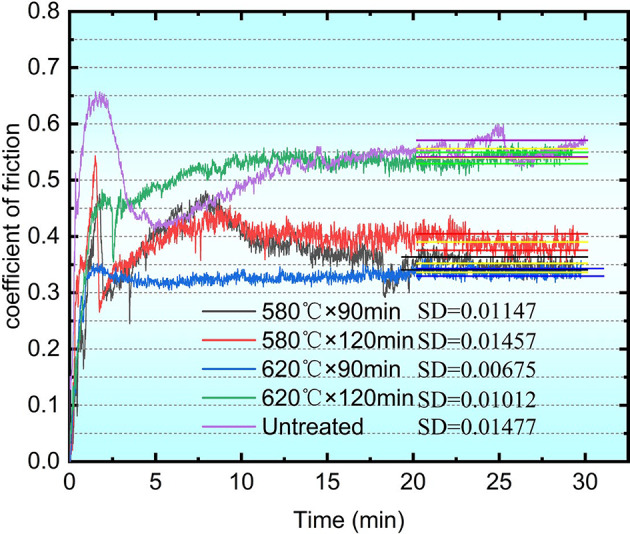
Comparison of friction coefficients under different QPQ process conditions. Different colors represent distinct process parameters. The yellow straight line at the end of each curve indicates the mean value, while the straight line matching the curve’s color represents the standard deviation.

The friction and wear performance test results for the QPQ-treated samples are presented in Fig. 6, with the friction coefficients and wear loss during the stable stage listed in Table 2. In the initial stage, all samples exhibited significant fluctuations in the friction coefficient due to high initial surface roughness. During the stable stage, QPQ treatment led to a substantial reduction in the friction coefficient and an improvement in wear resistance.

**Table 2:** Friction coefficient during the steady stage.

Process	Friction coefficient	Weight before wear /mg	Weight after wear /mg	Wear weight /mg
580℃×90min	0.36	13622	13619	3
580℃×120min	0.38	13669	13665	4
620℃×90min	0.33	13688	13686	2
620℃×120min	0.53	13526	13520	6
Untreated	0.56	12253	12246	7

At 580 °C, extending the holding time from 90 min to 120 min resulted in a slight increase in the coefficient of friction, from 0.36 to 0.38. Additionally, compared to untreated samples, wear loss decreased by 43%. At 620 °C, extending the holding time from 90 min to 120 min resulted in a significant increase in the coefficient of friction, from 0.33 to 0.53, approaching the value of the untreated sample. Meanwhile, compared to the untreated sample, wear loss decreased by only 14%. The untreated sample had a coefficient of friction of 0.56 and wear loss of 7 milligrams.

Under 580 °C conditions, extending the holding time slightly increased the friction coefficient and wear loss. However, at 620 °C × 120 min, the friction performance deteriorated markedly, with significant increases in the friction coefficient and wear loss, resulting in a severe decline in wear resistance that approached the untreated sample.

## Corrosion resistance

**Fig. 7:**
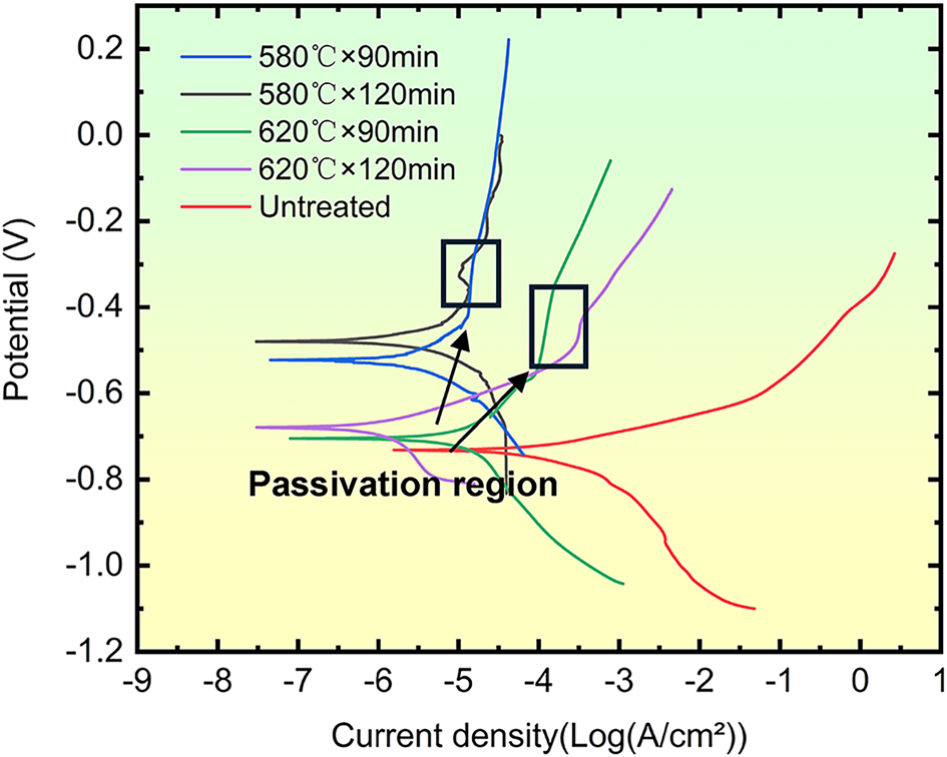
Potential-dependent polarization curves of QPQ-treated and untreated 42CrMo steel in a 3.5 wt% NaCl solution.

The polarization curves of QPQ-treated samples in a 3.5% NaCl solution are depicted in Fig. 7. The QPQ-treated samples exhibited a passive region in the anodic polarization curve, characterized by a stable or slightly decreasing current density with increasing potential within a specific range. In contrast, the untreated samples showed no passive region. Compared to the untreated samples, the corrosion potential of the QPQ-treated samples shifted positively, while the logarithm of corrosion current density decreased significantly.

The corrosion potentials (*E*_*corr*_*)* and logarithms of corrosion current densities (*Log*_*10*_*(I*_*corr*_*/cm²)*) under different QPQ treatment conditions were calculated using the extrapolation method from the polarization curves, as shown in Table 3. The untreated sample exhibited an *E*_*corr*_ of − 0.738 V and a *Log*_*10*_*(I*_*corr*_*/cm²)* of − 4.259, indicating the poorest corrosion resistance. All QPQ-treated samples demonstrated higher corrosion potentials and lower logarithms of corrosion current densities, confirming that the QPQ treatment improved the material’s electrochemical stability and overall corrosion resistance.

**Table 3:** E_corr_ and Log_10_(I_corr_/cm²) under different QPQ processes.

Process	*E*_corr_/ V	Log_10_(*I*_corr_/cm^2^)
580℃×90min	-0.525	-5.989
580℃×120min	-0.476	-6.242
620℃×90min	-0.704	-5.577
620℃×120min	-0.691	-6.053
Untreated	-0.738	-4.259

At a constant holding time, increasing the temperature resulted in a decrease in corrosion potential and an increase in the logarithm of the corrosion current density, indicating a reduction in corrosion resistance. For example, at a holding time of 90 min, raising the temperature from 580 °C to 620 °C caused *E*_*corr*_ to decrease from − 0.525 V to − 0.704 V and *Log*_*10*_*(I*_*corr*_*/cm²)* to increase from − 5.989 to − 5.577. At the same QPQ temperature, extending the holding time increased the corrosion potential and decreased the logarithm of corrosion current density, thereby enhancing corrosion resistance. For instance, at 620 °C, increasing the holding time from 90 to 120 min resulted in an *E*_*corr*_ increase from − 0.704 V to − 0.691 V and a *Log*_*10*_*(I*_*corr*_*/cm²)* decrease from − 5.577 to − 6.053.

**Fig. 8:**
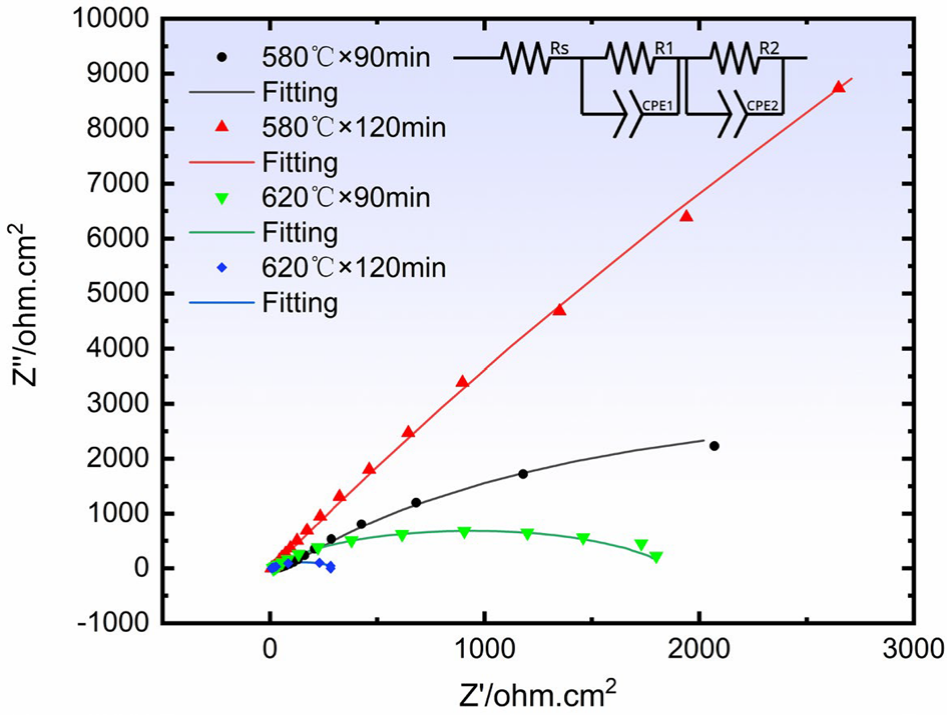
Nyquist impedance plots under different QPQ treatments. The circuit shown is an equivalent circuit model.

Figure 8 shows the Nyquist impedance plot for the QPQ sample. The depicted circuit is an equivalent circuit model in which Rs represents solution resistance and R1 represents interfacial charge transfer resistance. CPE1 denotes ideal capacitance, and R2 represents the capacitive behavior of the other interface. CPE2 is the second constant phase element representing the capacitive behavior of the other interface. The figure shows that the curvature radius at 580 °C is greater than that at 620 °C, indicating higher impedance and superior corrosion resistance. The 580 °C × 120 min treatment has the best corrosion resistance of the four samples. This demonstrates that extending the QPQ time further enhances the material’s corrosion resistance. As shown in Table 4, based on polarization resistance (Rp), these four samples’ corrosion resistance can be ranked as follows: 580 °C × 120 min > 580 °C × 90 min > 620 °C × 120 min > 620 °C × 90 min. The sample treated at 580 °C × 120 min exhibited the highest polarization resistance (Rp = 2643 Ω) and thus demonstrated the best corrosion resistance among the four samples. The sample treated at 620 °C × 90 min showed the poorest corrosion resistance with the lowest polarization resistance value (Rp = 288 Ω), indicating the fastest corrosion rate.

**Table 4:** QPQ sample EIS fitting Data.

Process	Rs / Ω	Rp / Ω	Error / χ²
580℃×90min	31.3	2040	8.969×10^-4^
580℃×120min	7.4	2643	9.333×10^-4^
620℃×90min	7.5	288	2.530×10^-3^
620℃×120min	13.6	1826	1.789×10^-3^

## Discussion

**Fig. 9:**
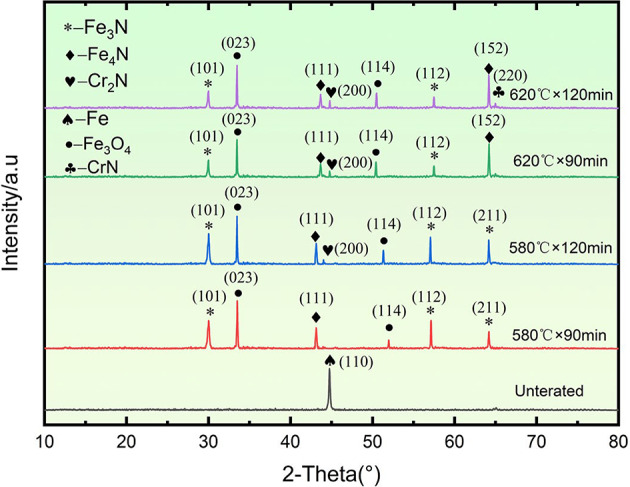
X-ray diffraction (XRD) patterns of 42CrMo samples under different QPQ treatment conditions. Untreated samples exhibit only the α-Fe phase.

The enhanced wear resistance achieved through QPQ treatment is attributed to forming a composite layer of Fe₂₋₃N, Fe₄N, and Fe_3_O_4_ nitrides on the surface, suppressing both adhesive and abrasive wear^29^. QPQ process parameters can regulate phase composition. Treatment at 580 °C for 90 minutes yields the highest nitrogen content (13.50 wt.%), favoring Fe₂₋₃N formation; whereas treatment at 620 °C promotes Cr₂N precipitation. Extending the 620 °C treatment time to 120 minutes results in distinct vein-like precipitates, increasing brittleness (Fig. 3). These nitrides are finely dispersed within the compound and diffusion layers, inducing lattice distortion and solid-solution dislocations. Combined with the surface Fe₃O₄ film, they enhance hardness and wear resistance^30–33^. Fe₂₋₃N and Cr₂N are hard but brittle; Cr₂N precipitates at grain boundaries and induces cracking, whereas γ’-Fe₄N exhibits a denser structure and superior toughness (Fig. 9). This confirms the minimum friction coefficient under the 620 °C × 90 min treatment condition. The 620 °C × 90 min treatment resulted in the best wear resistance (lowest friction and wear loss) under our test conditions, likely due to its dense and well-adhered Fe₄N compound layer. Simultaneously, Fick’s second law calculates the nitrogen diffusion coefficient at 620 °C as 9.25 × 10^−12^ m^2^/s—twice that at 580 °C (4.53 × 10^−12^ m^2^/s) (Table 1). Compared to the diffusion coefficient of ion nitriding at 5.36 × 10^−16^ m²/s^34^, the diffusion coefficient of the QPQ process is greater. This enables deeper penetration, making the material less susceptible to wear-through during prolonged abrasion. Although the 620 °C × 120 min treatment promotes Fe_4_N formation, it simultaneously weakens interlayer bonding strength and generates pores due to nitride decomposition, leading to an increased friction coefficient (Figs. 1 and 3; Table 2).

**Fig. 10:**
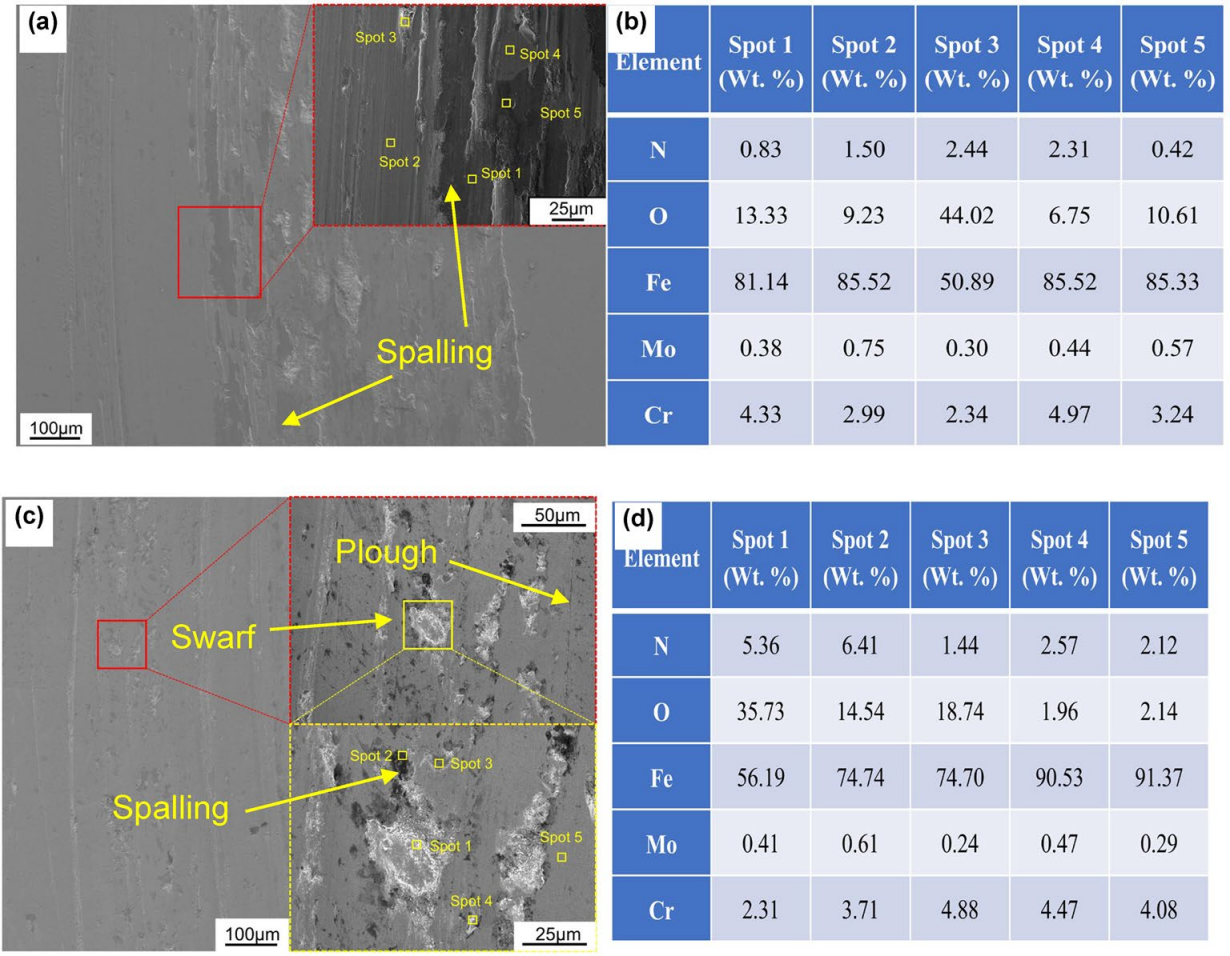
Scanning electron microscopy observation of surface morphology for QPQ-treated and untreated specimens: (**a**) Untreated specimen, (**b**) Specimen treated at 620 °C for 90 min; (**c**) Energy dispersive spectroscopy (EDS) data for untreated specimen, (**d**) EDS data for specimen treated at 620 °C for 90 min.

SEM and EDS analysis of wear morphology revealed ploughing grooves and spalling pits on the QPQ-treated samples, characteristic of tribotriplex wear induced by brittle Fe₂₋₃N and Cr₂N phase debris (Fig. 10). Surface spalling was significantly reduced on QPQ-treated specimens compared to untreated ones, with abrasive wear as the predominant form. Untreated specimens exhibited large-scale flaking dominated by adhesive wear^35,36^. EDS analysis of QPQ-treated wear debris and flaking pits showed elevated oxygen and nitrogen content, indicating oxide and nitride flaking. In contrast, untreated specimens suffered severe surface flaking due to the absence of nitride phases. Furthermore, the enhanced friction performance is partially attributed to the transformation of Fe₄N into lubricating FeO under frictional heat^37–40^. This demonstrates that wear resistance depends on the optimal balance between microstructure and toughness, rather than solely pursuing maximum hardness.

QPQ treatment enhances corrosion resistance through a dual-layer structure: an outer layer of dense Fe₃O₄ oxide film and an inner layer of chemically stable nitride compounds. This dual-layer structure blocks ion transport of corrosive media and alters surface reaction kinetics, shifting the corrosion process from “electrochemically active control” to “interface transport-limited control.” Corresponding electrochemical characteristics include a substantial decrease in corrosion current density on polarization curves (Table 3), accompanied by passivation phenomena in the anodic branch, as well as a significant increase in low-frequency impedance in impedance spectra (Table 4). This occurs because the interface is shielded by a continuous, dense nitride film, blocking ion transport pathways to the substrate. Concurrently, solid-solution nitrogen reacts with H⁺ ions while repelling Cl⁻ anions, elevating the substrate electrode potential and promoting repassivation^41–43^. The nitrogen-rich diffusion zone, acting as a gradient structure, increases the separation between the medium diffusion pathway and the electrochemically active sites from the substrate. This reduces local electrochemical inhomogeneity and suppresses pitting initiation.

However, when the temperature was increased to 620 °C, its corrosion resistance notably decreased compared to the 580 °C treatment, and extending the soaking time failed to improve this phenomenon. This deterioration stems from two factors: decomposition of the highly corrosion-resistant Fe₂₋₃N phase leads to surface porosity formation (as shown in Table 1; Fig. 3); and the generation of Cr₂N depletes chromium from the surrounding matrix, hindering the formation of a continuous passivation film^44,45^ (Figs. 3 and 9). Unlike wear resistance, which prioritizes “deep” nitride layers, corrosion resistance emphasizes the compactness of surface compound layers. Intense diffusion layers offer limited direct benefit to corrosion resistance. Although nitrogen exhibits a higher diffusion coefficient at elevated temperatures, resulting in deeper diffusion layers, the improvement in corrosion resistance is negligible. Extending the heat time at specific temperatures enhances compactness, leading to further corrosion resistance gains (Table 1).

This study demonstrates that the parameters of the QPQ process can be adjusted to meet various service requirements. For instance, a temperature of 620 °C for 90 min yielded excellent wear resistance, while a temperature of 580 °C for 120 min resulted in excellent corrosion resistance. These results suggest prioritizing wear resistance for load-bearing and friction-intensive applications and corrosion resistance for humid and corrosive environments when selecting a process. However, several limitations must be acknowledged. First, the experiments were conducted at two temperatures and two hold times, using a single salt-bath composition. Second, results may differ under other conditions. Additionally, only one alloy (42CrMo steel) was studied, so the findings may not apply to steels with different compositions or heat treatments. Additionally, wear and corrosion tests were performed under specific laboratory conditions that may not fully reflect real operating environments involving variable loads, temperatures, or media. Due to limited equipment, the underlying mechanisms could not be thoroughly investigated. These limitations indicate that conclusions should be applied cautiously beyond the present scope and that further studies are needed to validate and extend the observed trends.

### Experimental materials and methods

The experiments used commercially available 15 mm diameter 42CrMo bars, and their main chemical compositions are listed in Table 5.

**Table 5:** Chemical composition of 42CrMo material.

Element（wt.%）	C	Cr	Mo	Si	Fe
42CrMo	0.42	0.98	0.16	0.18	Bal.

**Fig. 11:**
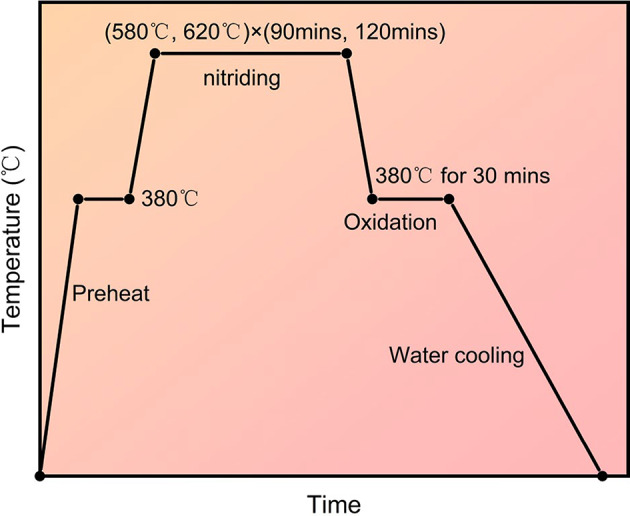
Process flowchart of 42CrMo QPQ treatment.

After quenching at 840 °C and tempering at 600 °C, 42CrMo steel undergoes surface modification via the QPQ process (as shown in Fig. 11). Before nitriding, the workpiece is immersed in a degreasing agent tank with a pH value ≥ 10 for 30–40 min, then rinsed with clean water to ensure oil-free surfaces. The workpiece was heated to 380 °C in a preheating furnace and held for 60 min. It was then transferred to a 580 °C/620°C nitriding furnace for 90 min/120 minutes. After nitriding, it was moved to a 380 °C oxidation furnace for holding for 30 min. Finally, perform water quenching. Prepare specimens measuring 15 mm × 5 mm for microstructure observation, surface hardness testing, diffusion layer depth measurement, and corrosion resistance evaluation; prepare specimens measuring 15 mm × 10 mm for friction and wear testing.

Metallographic samples were prepared by cold mounting, followed by sequential grinding with 180–2000 grit silicon carbide abrasive papers. Polishing was then performed on a P-1 polishing machine using W2.5 and W1.5 diamond polishing pastes until the surfaces were free of scratches. The polished surfaces were etched with a 4% nitric acid–ethanol solution (volume fraction). Microstructural observations and analyses were carried out using an RX50M optical microscope and an SU8000 scanning electron microscope (SEM). Phase identification was conducted using a Rigaku Ultima IV X-ray diffractometer (XRD) with a Cu target, a scanning rate of 5°/min, and a scanning range of 10°–80°.

Surface hardness and cross-sectional gradient hardness were measured using an HVC-5D1 Vickers hardness tester. Prior to surface hardness testing, the sample surfaces were ground with 800–1000 grit abrasive papers to remove the oxide and porous layers, followed by polishing. Samples for diffusion layer depth measurement were taken from the material cross-section and prepared using the same procedure as for metallographic samples, except that etching was omitted. Hardness testing was performed with a load of 0.2 kg (HV0.2) and a dwell time of 10 s. For gradient hardness measurements, the spacing between adjacent indentations was set to three times the diagonal length of the initial indentation. Measure the surface hardness of each sample six times and the gradient hardness three times.

Friction and wear performance were evaluated using a ball-on-disk dry sliding test at room temperature. The tests were performed on an MXS-01 friction and wear testing machine under a load of 10 N, a rotation diameter of 10 mm, a speed of 300 rpm, and a duration of 30 min. The test specimen serves as the lower plate, with GCr15 bearing steel balls acting as the counter-abrasion surface. These balls have a hardness of HRC 65 and a diameter of 10 millimeters.

Corrosion resistance was evaluated using a three-electrode system on a CHI electrochemical workstation (Shanghai Chenhua). A saturated calomel electrode (SCE) served as the reference electrode, and a platinum (Pt) electrode was used as the counter electrode. Prior to testing, the open-circuit potential (OCP) of the 42CrMo samples was measured. Polarization scans were performed over a range of OCP ± 500 mV at a scan rate of 0.5 mV/s.

The EIS test was evaluated using a three-electrode system on a CHI electrochemical workstation (Shanghai Chenhua). The test parameters are as follows: the high frequency is 100,000 Hz, the low frequency is 0.01 Hz, the sine wave amplitude is five mV, the frequency points are two points, and the applied voltage is the material’s open-circuit voltage.

## Data Availability

The data that support the findings of this study are available from the corresponding author upon reasonable request.
